# A Study of Some of the Rectal Disorders of Pregnant Women

**Published:** 1887-12

**Authors:** Edward Clark

**Affiliations:** Demonstrator of Anatomy, etc., Medical Department, Niagara University


Vol. XXVII. DECEMBER, 1887. No. 5

### Original Communications.

### *A STUDY OF SOME OF THE RECTAL DISORDERS OF PREGNANT WOMEN.**

By EDWARD CLARK, M. D.,

Demonstrator of Anatomy, etc., Medical Department, Niagara University.

Some time ago, I informed the Secretary of the Obstetrical Society, that, to-night, I would present a paper on "Hemorrhoids in Pregnancy," and a notice to that effect appears in the little pamphlet which has been issued to the members of this Club. After due consideration, I reached the conclusion, that the subject chosen was not comprehensive enough to enable me to write much concerning it that would interest the members of this Society. I have, therefore, taken the liberty to prepare a short paper on "Some of the Rectal Disorders in Women," not with the view of presenting anything new or original in this important field of study, but simply to refresh our minds by the discussion of certain affections, as they occur in females, many of which are, to a greater or less degree, caused or aggravated by the puerperal condition. The frequent occurrence of rectal diseases, and the undue amount of suffering and misery induced thereby, should serve to enkindle, in the mind of every intelligent

---

* Read before the Buffalo Obstetrical Society, September 27, 1887.

and conscientious physician, a desire to understand their pathology and treatment, but, unfortunately for himself as well as his patient, the general practitioner is not very fond of exploring the rectum. I say unfortunately for himself, for the reason that, in many cases, a physician has lost not a little well-earned reputation by failing to make a diagnosis, because he did not avail himself of the information which he might attain by a careful rectal examination. Many an unfortunate being has been allowed to suffer for weeks, months, or, perhaps, years, on account of mistakes in diagnosis, which might have been avoided by a careful exploration of the lower bowel. A striking illustration of this statement is found in the following interesting case, reported by Dr. Griffeth in the *Edinburgh Medical Journal*, which I take the liberty to quote in full:

"Mrs. G——, *aet.* twenty-five, mother of three children, the last being about four months old when I was first in attendance. I was called up to her on the night of June 18, 1876. She was suffering acute pain in the left side, which she could endure no longer. On examining the abdomen, I found a hard, irregular, exceedingly tender tumor, from which she was enduring great agony, and which was almost as large as an infant's head. I made no further examination that night, contenting myself with ordering her one-half grain morphia suppositories, to relieve not only the pain, but likewise the tenesmus and the passing of mucus. The discharge from the bowels was quite fluid, but distinctly fæcal; occasionally, a scybalous mass making its appearance. Next day, the morphia having taken good effect, I examined with the finger by the vagina, but could make out neither ovarine or uterine tumor. The sound *in utero* enabled me to make certain that there was no intra-uterine growth; but movement of the uterus, with the sound in the interior of it, was attended with the movement of the mass, which I found lay outside of the womb, yet connected to the left and upper portion of it—in fact, attached to it. I gave it as my opinion, that whatevcr the mass was, it was outside the uterus, and was adherent to it, and that it was not ovarian. I did not, however, express the opinion, at which I arrived after the above examinations, and after thoroughly exploring by the rectum, viz., that it was a case of impacted and accumulated fæces, which, having set up great irritation, had occasioned inflammation, effusion of lymph, and matting or glueing of the bowel to the left and upper portion or cornua of the uterus, that organ being still enlarged, its involution after delivery being not yet completed, probably owing to the irritation, inflammation, and subsequent adhesion to which I have referred. Taking this view of the case, I purged freely and continuously for some days, till at length, after the lapse of six weeks, I had the satisfaction of hearing from my patient, (for I did not attend her continuously during the period,) that the tumor was all gone, and she was quite well—facts I verified by careful manipulation when she last visited me. The iodide of potassium had been combined with the aperients, as had also anodynes—the former in hope of dissolving adhesions, the latter with a view to ease pain. I would add, to show the difficulties which sometimes behedge the diagnosis in these cases, that this patient had previously had pronounced to her, by three medical men, that operation alone (gastrotomy) could do her any good, and of this she had a mortal dread, so that, all through, I buoyed her up with the hope that the knife might never be required. The swelling had commenced to be noticed about twelve or fourteen days after the birth of her child, was chiefly confined to the left side, though sometimes it seemed to enlarge and extend higher up and across the middle line toward the right, and was so large, that it was as though she was at her full time, and, when walking even across her room, she required a towel to support the abdomen; at other times, it would subside, preserving, however, the same shape; these alterations in size were synchronous with the action of the bowels, and gave me a valuable clue. The agony had been very great, and she told me nothing had relieved her for any length of time till she had used the morphia suppositories. At no period was there a discharge of matter indicative of any internal abscess; nor any flux of water, either into the abdominal cavity, or into the bladder, or any way externally, which would demonstrate the existence and rupture of an ovarian or other cystic growth; therefore, the only diagnosis at which I could arrive, was that the bowels had become blocked during the confinement period, and had not emptied themselves fully; that an accumulation occurred and became greater and greater, being, however, occasionally partially lessened by the aperient action of the bowels themselves, which accounted for the diminution of, and subsidence that had been noticed in the swelling."

I have quoted this case in full, on account of its very interesting character, and also to show what may be accomplished in such cases by a thorough rectal examination. Here was a woman consigned by three medical men to all the dangers of gastrotomy, because they failed to make a correct diagnosis, I believe, for the reason that they did not make a sufficiently careful rectal exploration. At this critical point, a surgeon comes in, and relieves the patient of all her anxiety, and cures her in a few weeks. He possesses a "mind imbued with the true professional spirit," and has no more scruples against entering the rectum of his patient than the average gynecologist or obstetrician has against entering a woman's vagina. Many cases, similar to the one here reported, might be cited to prove that physicians do not, will not, or cannot, make a proper rectal examination. I can call vividly to mind a case of ischio-rectal abscess, which I saw, a few months since, at the U. S. Marine Hospital, at Stapleton, Staten Island. The man was reduced almost to a skeleton by reason of chills, sweats, hectic, and the excruciating pains which he had undergone while under the care of a private practitioner, who never made a rectal examination in his case, although the patient continually referred his trouble to that organ. The man entered the hospital in an extremely nervous, debilitated condition; a rectal examination was made, and the difficulty at once recognized. This man believed that he was suffering from cancer. A short time ago, a physician of this city related to me an account of a case of ischio-rectal abscess, which he saw in consultation with another practitioner. This patient complained constantly of pain in and about the ano-rectal region, had chills, sweats, etc., yet no rectal examination was made. The case went on from bad to worse, and still no diagnosis, until at last, about the time my informant saw the patient, a perineal fistula had formed. Nature had succeeded in finding an outlet for the pent-up matter, but not until the patient's system had become so much contaminated with septic material, that efforts at recuperation were vain ; the unfortunate patient soon being released from his sufferings by death from pyæmia, with all its concomitant phenomena. I trust I may be pardoned for this digression from my subject proper, but it seems necessary to relate some cases to substantiate my assertions relative to the indifference and carelessness of physicians, as regards the diagnosis of rectal affections.

Among the many rectal disorders, which are, more or less, influenced by pregnancy and parturition, may be mentioned hemorrhoids, fæcal fistulæ, ulceration and stricture, and neuralgia of the rectum—or irritable rectum, as it is sometimes called.

A word or two concerning some of these affections is all that the scope of this paper will allow.

Every physician is well aware that hemorrhoidal tumors are quite common in the latter months of pregnancy, and that they frequently give rise to a vast amount of discomfort. When of large size, they sometimes become strangulated, and the excessive pain thus produced has been known to cause abortion. These piles in pregnancy are produced largely by the pressure of the gravid uterus on the pelvic veins, and the constipated condition usually found in the pregnant state. The veins of the pelvis are also normally much more engorged with blood during gestation than at other times. The extreme tension to which these hemorrhoidal growths are sometimes subjected by pressure, combined with constipation, may cause them to bleed. This, of course, brings temporary relief to the patient, but you will sometimes meet with cases in which a large rupture has occurred in the vessels composing the growth, from which an increasing and sometimes alarming hemorrhage may occur, which may greatly lower your patient's vitality, and expose her to the various nervous and other derangements consequent upon the anæmic condition, unless prompt measures for the arrest of the hemorrhage be instituted. Many women, in the later months of gestation, are troubled, more or less, with irritability of the bladder. This, of course, is due, in a large number of instances, to the pressure exercised, the enlarged uterus; and I believe that many physicians are prone to recognize this as the only cause of vesical irritation late in pregnancy. If your patients are carefully questioned on this subject, many of them will admit they are suffering from constipation and hemorrhoidal growths, the existence of which they have kept concealed from their medical adviser from motives of false delicacy. Many women have been treated in vain for this vesical tenesmus, for the reason that its cause, the hemorrhoids, has been ignored, while vigorous therapeutic measures have been resorted to, to remove what is only a symptomatic or reflex phenomenon. Every practitioner is well aware of the irritable condition of the bladder, accompanied sometimes by urinary retention, which follows on abnormal growths and operations in the ano-rectal and perineal regions; and, unless one has his eyes open to the fact, he will be found frequently treating the effect, and ignoring the cause.

In the pregnant female, external hemorrhoids are much more frequently met with than internal, and the treatment, aside from occasional incisions, is largely dietetic and medicinal. Internal hemorrhoids, which bleed or become prolapsed and strangulated, demand surgical interference for their relief and cure. For the internal bleeding pile, nothing, perhaps, answers better than the occasional application of nitric acid. I do not think I would hesitate to operate on a pregnant woman with large prolapsing hemorrhoids, for, other things being equal, I do not think premature labor would follow the operation any more frequently than it would result from the irritable and painful hemorrhoids, if left undisturbed. For the large prolapsing hemorrhoids, I would resort preferably to the clamp and cautery operation which has been so successful in the hands of my friend, Dr. Kelsey, of New York; or the method of crushing, advocated by Pollock, and now extensively practiced by Allingham. I have a very high opinion of the method of exsection, recently so ably advanced by Whitehead, and I believe it would be a most eligible procedure in these cases, but, as yet, I have had no experience with it. I prefer the operative procedures above named to the ligature, and injections of caustics, astringents, etc., for the reasons that, as a rule, I believe they are followed by much less pain and reflex disturbance. Moreover, the method of treating hemorrhoids by injection is not, in my opinion, a very scientific operation, and while, perhaps, in a few selected cases, it is productive of favorable results, its indiscriminate employment by many who now resort to it is prolific of much mischief. A few words concerning some of the other affections named. Of fæcal impaction, which occurs not infrequently in pregnancy, I shall say nothing, for the case I have quoted in full from Dr. Griffeth, embodies all that is most interesting in the study of that condition. Ulceration and stricture occur more frequently in women than in men, but just how much etiological importance is to be attached to maternity in these conditions, is not fully determined.

Brodie, Allingham, Curling, Molliere, Kelsey, Van Buren, and other good authorities on rectal diseases, recognize the fact that a bruising of the rectal wall, between the child's head and the sacrum, produces a traumatism, resulting, sometimes, in stricture and ulceration. Many authorities seek to explain, in this way, the greater frequency of ulceration and stricture in the female sex. Van Buren says: "I had once, in my wards in the New York Hospital, a healthy, respectable woman of thirty-five, with an extensive surface ulceration surrounding the gut, for two or three inches above the sphincter, which followed a long, hard labor. The ulceration finally got well, but its cicatrization resulted in the formation of a stricture. This case left in my mind a very strong impression, that the constant liability to injury, sustained in this way, must go far toward explaining the greater frequency of ulceration and stricture of the rectum in women."

Brodie mentions such cases in his writings, and Curling gives a table of twenty-eight cases of stricture of the rectum, twenty of which were in women, and in nine of them the disease commenced after labor, in some instances being distinctly attributed to an injury at that time. Undoubtedly, in the majority of cases in which ulceration and consequent stricture follow a prolonged, hard labor, there is present in the patient some accessory cause, in the shape of anæmia, or the strumous and syphilitic diathesis, which prevent a prompt repair of the solutions of continuity, produced by a difficult parturition.

The processes of repair in a healthy, robust woman, are generally so active, that a prompt recovery soon follows any bruising of the rectal walls, as well as of other parts, while in one who is weakened by any of the abnormal conditions above named, the process of repair is retarded to such an extent, that the patient is exposed to that chronic ulceration and inflammation of the mucus and sub-mucous areolar tissues of the rectum, which sooner or later ends in the formation of stricture. These changes, together with the contractions caused by the healing of ulcers, are recognized as the principal pathological factors in producing this affection. Fibroid tumors of the uterus, both intra-uterine and those which have been extruded into the vagina, may cause ulceration and stricture by pressure on the rectum.

Prolonged pressure from the child's head in protracted labor, may cause a complete loss of vitality, resulting in sloughing of the rectal wall at some points, thus producing another affection occasionally met with, viz., fæcal fistula, which I shall treat of very briefly. These fistulæ are less frequently met with than the urinary, and several varieties are described in the text-books. The most common form is the recto-vaginal, and the only one that concerns us in a paper of this character. In addition to pressure from the fœtal head, we must recognize badly-fitting pessaries, and the improper use of instruments during delivery, as among the etiological agencies of this form of fistula. Perhaps, the most common, and frequently the only, symptom which admonishes the patient of the presence of this disagreeable malady, is an offensive discharge from the vagina. The quantity will, of course, depend to a certain extent upon the size of the fistulous opening, but, be it ever so small, the poor patient's existence is a wretched one, indeed, owing to the very offensive odor; the presence of which debars her, to a great extent, from entering into many of the social enjoyments of life. Fortunately, this disagreeable affection is very amenable to the resources of surgical art, and the unfortunate sufferers may easily be relieved of a condition which, to all women of delicate and refined feelings, must be a heavy cross to bear, rendering them, as it often does, repugnant to their friends, their families and themselves. One can scarcely fail in making a proper diagnosis of the affection. The presence of the fistulous opening can generally be determined by a visual examination alone. Should this fail, the rectum may be filled with milk, or some artificially-colored fluid, and its escape into the vagina will reveal the location of the diseased spot. Spontaneous recovery sometimes occurs in these cases, but it is not always wise to wait for this result, when the simple operation of denudation and suturing is almost absolutely certain to effect a cure in the large majority of cases.

Before operating, the bowels should be thoroughly emptied by cathartics and an enema, and the sphincter ani muscle should be temporarily paralyzed by forcible stretching with the fingers. The bowels should be confined for a week or more after operating, by means of opiates, and, when first allowed to move, laxatives or an enema should be used, to prevent too much tension on the newly-healed wound.

